# Induction of LTM following an Insulin Injection

**DOI:** 10.1523/ENEURO.0088-20.2020

**Published:** 2020-04-27

**Authors:** Yuki Totani, Junko Nakai, Varvara E. Dyakonova, Ken Lukowiak, Manabu Sakakibara, Etsuro Ito

**Affiliations:** 1 Department of Biology, Waseda University, Tokyo 162-8480, Japan; 2 Koltzov Institute of Developmental Biology, Russian Academy of Sciences, Moscow 119334, Russia; 3 Hotchkiss Brain Institute, University of Calgary, Calgary, Alberta T2N 4N1, Canada; 4 Research Organization for Nano & Life Innovation, Waseda University, Tokyo 162-8480, Japan; 5 Graduate Institute of Medicine, School of Medicine, Kaohsiung Medical University, Kaohsiung 80756, Taiwan

**Keywords:** conditioned taste aversion, food deprivation, glucose, insulin, *Lymnaea*

## Abstract

The pond snail *Lymnaea stagnalis* learns conditioned taste aversion (CTA) and consolidates it into long-term memory (LTM). One-day food-deprived snails (day 1 snails) show the best CTA learning and memory, whereas more severely food-deprived snails (5 d) do not express good memory. However, previous studies showed that CTA-LTM was indeed formed in 5-d food-deprived snails (day 5 snails), but its recall was prevented by the effects of food deprivation. CTA-LTM recall in day 5 snails was expressed following 7 d of feeding and then 1 d of food deprivation (day 13 snails). In the present study, we thus hypothesized that memory recall occurs because day 13 snails are in an optimal internal state. One day of food deprivation before the memory test in day 13 snails increased the mRNA level of molluscan insulin-related peptide (MIP) in the CNS. Thus, we further hypothesized that an injection of insulin into day 5 snails following seven additional days with access to food (day 12 snails) activates CTA neurons and mimics the food deprivation state before the memory test in day 13 snails. Day 12 snails injected with insulin could recall the memory. In addition, the simultaneous injection of an anti-insulin receptor antibody and insulin into day 12 snails did not allow memory recall. Insulin injection also decreased the hemolymph glucose concentration. Together, the results suggest that an optimal internal state (i.e., a spike in insulin release and specific glucose levels) are necessary for LTM recall following CTA training in snails.

## Visual Abstract

**Figure:**
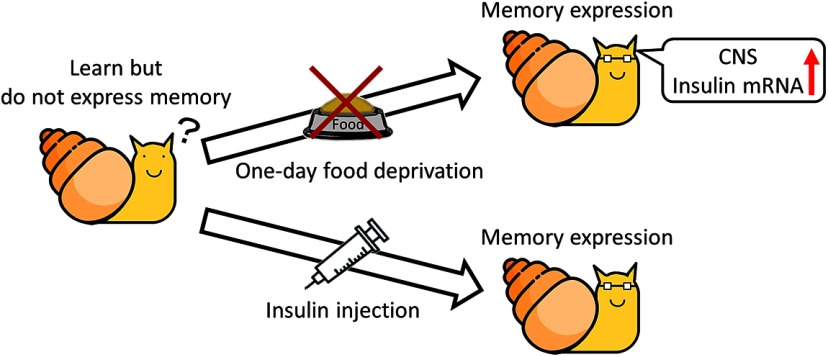

## Significance Statement

When snails are trained for conditioned taste aversion (CTA) in a relatively long food-deprived state, they express long-term memory (LTM) if food is deprived again before the memory test. We hypothesized that there is an optimal state, such as increased insulin levels and reduced glucose levels in the hemolymph, in snails that allows memory recall. An insulin injection instead of food deprivation before the memory test caused the optimal internal state to occur in the snails that initially did not express CTA-LTM. Insulin directly modulates synaptic transmission in CNS neurons and alters learning and memory.

## Introduction

Nutrition affects cognitive function not only in humans (Gailliot and Baumeister, 2007; Morley, 2014) but also in other animals (Xia et al., 1997; Swinton et al., 2018). Insulin and insulin-like peptides decrease circulating glucose levels in blood and hemolymph (Horn et al., 1998; Kim and Rulifson, 2004; Zheng and Greenway, 2012). Further, insulin signaling is a candidate for sensing nutritional status (Sjøberg et al., 2017). Moreover, recent studies have revealed that insulin and related peptides are strongly involved in cognitive functioning (Cholerton et al., 2013; Sasakura and Mori, 2013; Akinola, 2016).

The pond snail *Lymnaea stagnalis* can learn conditioned taste aversion (CTA) and consolidate it into long-term memory (LTM; Kojima et al., 1996, 1998; Aonuma et al., 2018a,b). To produce CTA in *Lymnaea*, an appetitive stimulus (e.g., sucrose) is used as the conditioned stimulus (CS). Application of the CS to the lips increases the feeding response. An aversive stimulus (e.g., KCl or electric shock) is used as the unconditioned stimulus (US). Application of the US to the snails inhibits feeding behavior. After repeated pairings of the CS with the US, the CS no longer elicits a feeding. CTA-LTM persists for more than a month (Kojima et al., 1996). Nutritional status significantly affects CTA learning and memory in snails and insulin plays an important role in this (Hatakeyama et al., 2013; Kojima et al., 2015; Totani et al., 2019). One-day food-deprived snails (i.e., day 1 snails) show the best CTA learning and memory-forming ability, whereas more severely food-deprived snails (i.e., 5-d food-deprived snails: day 5 snails) do not express the CTA memory phenotype (Sugai et al., 2007; Mita et al., 2014a,b). However, the ability to recall the CTA memory in day 5 snails occurred following feeding for 7 d and then being placed into the day 1 state. That is, when day 5 snails were fed and then food deprived for 1 d, they recalled the memory. Thus, day 5 snails following training need to eat and then undergo the 1-d food deprivation before the memory test to recall CTA-LTM (Ito et al., 2015).

During CTA training, it was previously shown that the expression of some of molluscan insulin-related peptide (MIP) genes were upregulated (Azami et al., 2006). Additionally, when either MIPs or mammalian insulin were superfused over the isolated CNS, long-term synaptic enhancement occurred between neurons mediating CTA (Murakami et al., 2013a). Finally, the injection of insulin into freely behaving *Lymnaea* enhances CTA-LTM (Murakami et al., 2013b; Mita et al., 2014a,b). We thus hypothesized that when insulin is administrated to day 5 snails which were then given 7 d of *ad libitum* access to food (day 12 snails) before the memory test, insulin activates CTA-related neurons and reduces the hemolymph glucose concentration to create an internal state conducive for LTM recall. That is, LTM was formed in day 5 snails but could not be recalled due to a non-optimal internal state. Thus, for CTA-LTM to be expressed, an optimal internal state must be achieved to allow recall.

## Materials and Methods

### Snails

Specimens of *L. stagnalis* with a 20- to 25-mm shell length obtained from our snail-rearing facility (original stocks from Vrije Universiteit Amsterdam) were used in the present study. All snails were maintained in dechlorinated tap water (i.e., pond water) under a 12/12 h light/dark cycle at 20–23°C and fed *ad libitum* on turnip leaves (*Brassica rapa var. peruviridis* known as *Komatsuna* in Japanese). Food deprivation was conducted for 1 d (referred to as day 1 snails) or 5 d (referred to as day 5 snails) before the CTA training. Day 12 snails first followed the day 5 snail procedure, and they were then given 7 d of *ad libitum* access to food before being tested on day 12. Day 13 snails were similar to day 12 snails, except they were food deprived for 1 d following 7 d of food access. It needs to be noted that when a cohort of snails received two sets of the 5-d food-deprivation procedure, the death ratio increased significantly. Thus, such a sever state of food deprivation leads to an extreme stressful state in these snails.

### Protocol of CTA training

We used an automatic training apparatus (Takigami et al., 2016; Sunada et al., 2017), with slight modifications (Totani et al., 2020). The main difference from the previous studies was the US used. In the present study, we used a KCl solution instead of electric shock. The experimental system consisted of five independent training chambers with a 50-ml test chamber flowing continuous water stream (3.3 ml/s), and a snail placed in each of the test chambers was physically fixed at the anterior and posterior parts of a shell with a hand-made clip not to change their positions. Snails were stimulated with 100 mm sucrose (the CS) and 200 mm KCl (the US) by flowing it into the test chambers for 15 s. Fluids were drained from the chamber via an overflow pathway.

**Figure 1.**
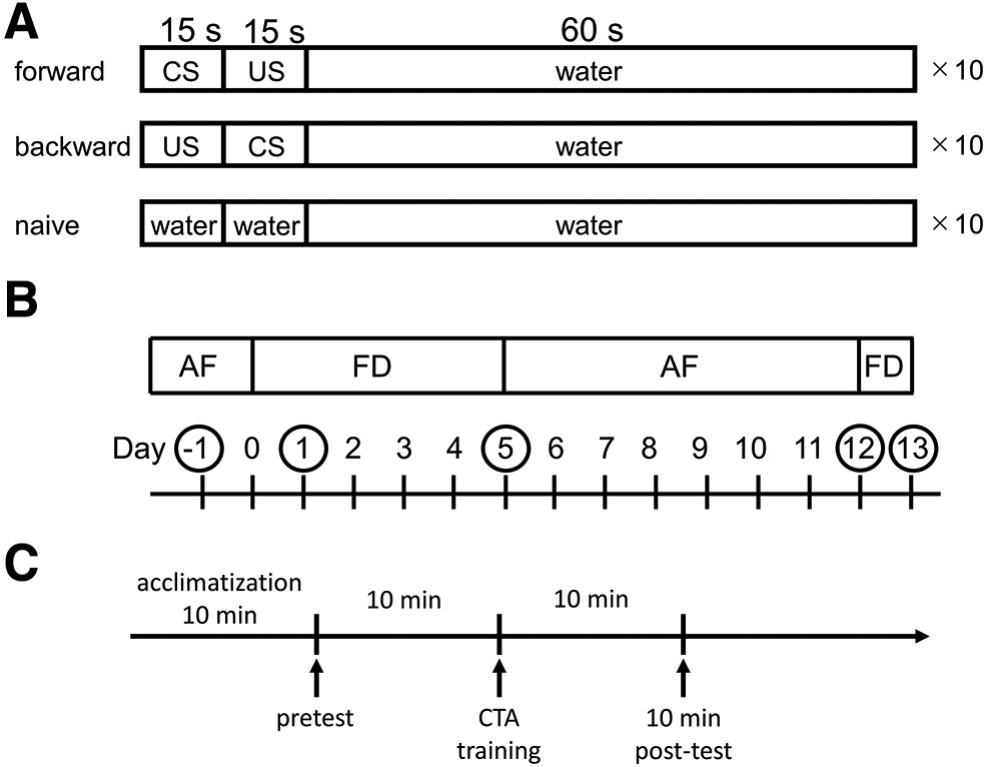
The CTA training procedures and time lines of food deprivation. ***A***, All snails were first given a pretest 10 min before the training. In this observation period (1 min), the feeding responses (i.e., number of bites) were counted following a 15-s application of 100 mm sucrose (the CS) to the lip of the snail. For taste aversion training, the 100 mm sucrose CS was paired with the 200 mm KCl US. The interstimulus interval was 15 s between the onset of the CS and US. A 90-s ITI was interposed between each pairing of the CS-US. Snails received 10 paired CS-US trials on a single day. Controls included a backward-conditioned (US-CS) group and a naive group to validate associative learning. For the naive control group, only water was applied to the lips instead of the CS and US. In the post-test sessions, snails were again challenged with the CS, and the number of bites was recorded in the 1-min interval after a 15-s application of the CS. ***B***, Food-deprivation status was defined in the following manner: (1) the day when snails began food deprivation is called day 0; (2) day −1 snails had *ad libitum* access to food (AF), that is, they were not food deprived (FD); (3) day 1 and day 5 snails were food deprived for 1 and 5 d, respectively, after food deprivation, had *ad libitum* access to food (AF); (4) day 12 snails had *ad libitum* access to food for 7 d following training on day 5; and (5) day 13 snails were food deprived for an additional 1 d following 7 d of *ad libitum* access to food. ***C***, Snails were first placed in the test chamber for 10 min to acclimate to the training apparatus. Following the acclimatization, a pretest session was performed, and 10 min after the pretest, CTA training was performed. After CTA training, several post-test sessions were performed to verify the memory expression.

The number of bites was recorded as a pretest in the 1-min interval in the test chamber after the 15-s application of the CS. After the pretest, 10 paired CS-US were applied with the intertrial interval (ITI) of 90 s. Immediately after the CS presentation (15 s), the US was applied for 15 s. Control experiments were performed in a backward-conditioned (US-CS) group and a naive group to demonstrate that associative learning occurred. For the naive group, only tap water was flowed on the snails instead of the CS and US. In the post-test sessions (i.e., the memory test), snails were again challenged with the CS, and the number of bites was counted in the 1-min interval in the test chamber after the 15-s application of the CS. After the 10 min post-test, snails were returned to their home aquarium and allowed *ad libitum* access to food. All the behavioral experiments were done in the morning (Wagatsuma et al., 2004). The time schedule of these protocols is shown in Figure 1*A*,*C*.

We defined the food-deprivation status of the snails in the following manner: (1) snails that are eating food are called “eating”; (2) the day when snails begin food deprivation is called “day 0”; (3) “day −1” snails are thus fed snails but they are not eating food; (4) “day 1” snails are the snails which were deprived of food for 1 d; (5) “day 5” snails were food deprived for 5 d; (6) “day 12” snails are food deprived for 5 d followed by an *ad libitum* access to food for 7 d; and (7) “day 13” snails are food deprived for 5 d followed by an *ad libitum* access to food for 7 d and then food deprived for 1 d (Fig. 1*B*).

Based on previous findings, a performance boundary in the memory test sessions was set to distinguish between good and poor learners (Sugai et al., 2007; Mita et al., 2014a,b). A snail possessing good learning and good LTM does not bite following the presentation of CS. However, some snails open their mouths by chance in the absence of any delivered stimulus (Kojima et al., 1996). Such spontaneous openings occur at a rate of about one per minute. Thus, a good learner following the CS-US pairings was defined as a snail that made zero to one bite per minute during the post-test sessions in response to the presentation of CS. Poor learners were defined as snails that made more than or equal to two bites per minute in response to the CS during the post-test sessions (Mita et al., 2014a,b).

### Real-time PCR

Total RNA was isolated from snails’ CNS using the RNeasy mini kit including RNase-free DNase set (QIAGEN). cDNA was generated by using Quantitect Reverse Transcription kit (QIAGEN) or ReverTra Ace (Toyobo) according to the reaction protocol. The comparative quantification method of real-time PCR for mRNAs of MIP I, MIP II, and MIP receptor (MIPR) in *Lymnaea* were performed using a BrightGreen 5× qPCR MasterMix-ROX (Applied Biological Materials). The mRNA levels were normalized to those of heat shock protein 40 (HSP40) and glutathione peroxidase (GPx). The primer sequences are shown in Table 1. Efficiency values for the real-time PCR primers ranged between 90% and 110% (*R*^2^ = 0.95–0.99). The PCR conditions were as follows: one cycle at 95°C for 20 s, followed by 40 cycles of denaturation at 95°C for 3 s; annealing at 60°C for 30 s. Melting curve analysis was performed from 60°C to 95°C with a heating rate of 0.3°C/s. Data were recorded on a StepOnePlus Instrument using StepOne Software version 2.3 (ThermoFisher Scientific).

**Table 1:** The primer sequences for real-time PCR amplification of specific *Lymnaea stagnalis* genes

Gene name	Accession number		Primer sequence	Product size (bp)
MIP I	X06983.1	Forward	AGCGCTTTGACCTACCTGAC	81
		Reverse	ACTCAGTGTGCACGGTTTCA	
MIP II	X59302.1	Forward	AGAGGGCCAATCATCTTGCAG	77
		Reverse	GGAAGCCAGCCAAATTCGAG	
MIPR	X84994.1	Forward	TGACCAGACTGGAACCTGGA	106
		Reverse	GGTGGACGTGGCACTATGAA	
HSP40	DQ278442.1	Forward	GGTCTTGAATCCTGATGGACA	104
		Reverse	CTTTGGGGAAGGTTATTTTGG	
GPx	FJ418796.1	Forward	TGTAAACGGGACGGAGATTC	118
		Reverse	GATCTCGTTTTCCCCATTCA	

### Drug injection

Instead of 1-d food deprivation in some day 13 snails, snails were injected with either bovine insulin prepared (Sigma-Aldrich), mouse monoclonal [47–9] antibody to human insulin receptor α-subunit (ab982; Abcam) or *Lymnaea* saline with HCl (vehicle control group), whose final concentration in the body was estimated as 100 nm, 2.56 μg/ml (17.5 nm), or 120 nm, respectively. To examine the effect of MIPs on hemolymph glucose concentration, MIPs purified partially by Murakami et al. (2013a), were used at the final concentration of 50 nm. Drugs were injected into abdominal cavity of the snail using a microliter syringe (Hamilton Company). The mouse monoclonal [47–9] antibody blocks the binding between insulin and insulin receptors (Soos et al., 1986; Taylor et al., 1987).

Previous experiments demonstrated that this insulin receptor antibody can bind *Lymnaea* MIPR (Murakami et al., 2013a,b; Mita et al., 2014b). This is because the binding site of *Lymnaea* MIPR is well conserved across phyla. For example, in comparison with mammalian insulin receptors (accession numbers: CAA59353 for *Lymnaea*, AAA59174 for humans, and AAA39318 for mice; Jonas et al., 1996) the homology is 34% for the whole amino acid sequences; 56% for the ligand-binding domain 1 (L1 domain); and 33% for the ligand-binding domain 2 (L2 domain) between *Lymnaea*, humans, and mice. Although the three-dimensional structures of MIPs have not yet been elucidated, previous studies using another mollusk, *Aplysia*, have also demonstrated that application of bovine insulin activated the bag cell-neuron insulin receptor by stimulating its autophosphorylation on tyrosine residues (Jonas et al., 1996) and evoked egg-laying hormone secretion (Jonas et al., 1997).

In the procedure of drug application, the total volume of injection solution was 40 μl. The calculated volume of hemolymph in a snail with a 20-mm shell length was 0.4 ml (Sugai et al., 2006). One hour after injection of the insulin or antibody, the post-test for LTM was performed.

### Measurement of hemolymph glucose concentration

To measure the hemolymph glucose concentration, the pond water surrounding the snail was blotted up with absorbent paper. The snail was then given a poke with a needle, causing it to retract into its shell and expel hemolymph through its renal pore. The glucose hemolymph concentration was measured using a mutarotase-glucose oxidase assay kit (Glucose C2; Fujifilm Wako Pure Chemical Corporation). The measurements were performed according to the manufacturer’s manual with slight modification. We used 10 times the amount of sample solution compared with the original procedure to increase the sensitivity. *R*^2^ value of the calibration curve was 0.99 or more and the spike-recovery-test ratio was 102% (*n* = 3, SEM = ±2.0).

### Statistics

The data are expressed as mean ± SEM, with statistically significant at *p *<* *0.05. Comparison between two groups was analyzed using Welch’s *t* test. Data from multiple groups were analyzed using one-way ANOVA or two-way ANOVA followed by a *post hoc* Holm’s test. The computer software used was R (version 3.3.1; https://www.r-project.org/).

## Results

### Memory retrieval in severely food-deprived snails

**Figure 2.**
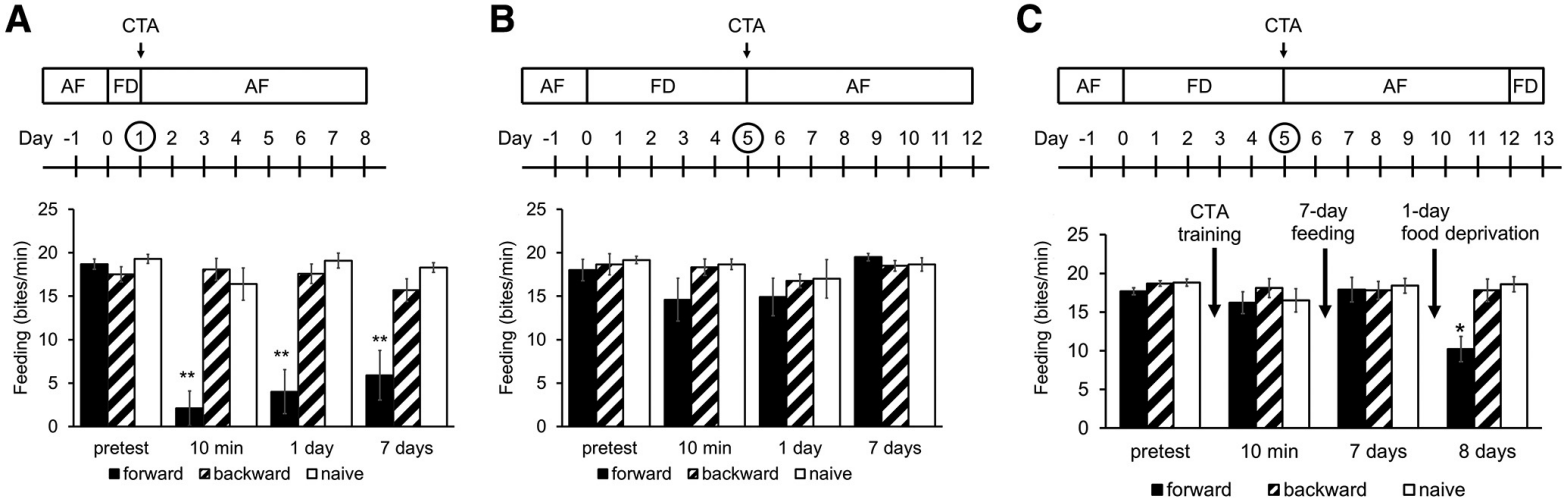
Memory formation in day 1, day 5, and day 13 cohorts of snails. The experimental schedule for each cohort of snails is shown at the top of figures. ***A***, The number of bites elicited by the CS (a 100 mm sucrose solution) in 1 min in the pretest session and the post-test sessions for day 1 snails are shown. The feeding response to the CS in the forward conditioned group was significantly reduced at the three memory test sessions (10 min, 1 d, and 7 d), compared with those observed for the backward-conditioned and naive control snails (two-way ANOVA, *n* = 10 each, *F*_(6,81)_ = 8.79, *p *<* *0.0001 for interaction, *F*_(2,81)_ = 29.5, *p *<* *0.0001 for conditioning, *F*_(3,81)_ = 11.6, *p *<* *0.0001 for test, Holm *post hoc* test **p <* *0.01). ***B***, As in ***A*** except the training and testing were performed on the day 5 snails (5 d of food deprivation before CTA training). Learning and memory are not expressed on the three post-training tests (10 min, 1 d, and 7 d; two-way ANOVA, *n* = at least 6, *F*_(6,57)_ = 0.50, *p *=* *0.80 for interaction, *F*_(2,57)_ = 1.18, *p *=* *0.33 for conditioning, *F*_(3,57)_ = 1.47, *p *=* *0.23 for test). ***C***, As in ***A***, ***B*** except that after the 10-min memory test snails were given *ad libitum* access to food for 7 d. Snails were thus tested for memory following this 7-d period. No LTM was observed. Finally, snails were given a single day of food deprivation, and memory was tested the following day. LTM was now observed (i.e., day 13 snails; two-way ANOVA, *n* = 10 each, *F*_(6,81)_ = 2.27, *p *=* *0.045 for interaction, *F*_(2,81)_ = 4.29, *p *=* *0.024 for conditioning, *F*_(3,81)_ = 2.41, *p *=* *0.073 for test, Holm *post hoc* test *p =* *0.031 for forward vs backward; *p =* *0.024 for forward vs naive). The error bars indicate SEM.

Previous studies showed that the feeding response to the CS was significantly suppressed in CTA-trained snails (Ito et al., 2017). The memory scores of CTA depended on the feeding status of the snails before the CTA training (Ito et al., 2015). Here, we examined the relationship between CTA behavioral changes and feeding status again. This is necessary because the training procedure used here was somewhat different from the previous one used (i.e., Ito et al., 2015). Snails with 1-d food deprivation (i.e., day 1 snails) showed the best learning score (*p *<* *0.0001; Fig. 2*A*). However, snails that were food deprived for 5 d (i.e., day 5 snails) did not express memory when tested 24 h after CTA training (Fig. 2*B*). Additionally, if day 5 snails were then given access to food for 7 d following the 24 h memory test, LTM was not observed on the memory test on day 12 (i.e., day 12 snails, 7-d post-test in Fig. 2*B*). Finally, if the procedure used in Figure 2*B* was modified by food-depriving day 12 snails for 1 d following 7 d of *ad libitum* feeding, LTM was observed on day 13 (Fig. 2*C*). These snails expressed CTA-LTM compared with those observed for the backward-conditioned and naive control snails (*p *=* *0.031 for forward vs backward; *p *=* *0.024 for forward vs naïve; i.e., day 13 snails, 8-d post-test in Fig. 2*C*).

### Upregulation of MIP II during short food deprivation before memory test

**Figure 3.**
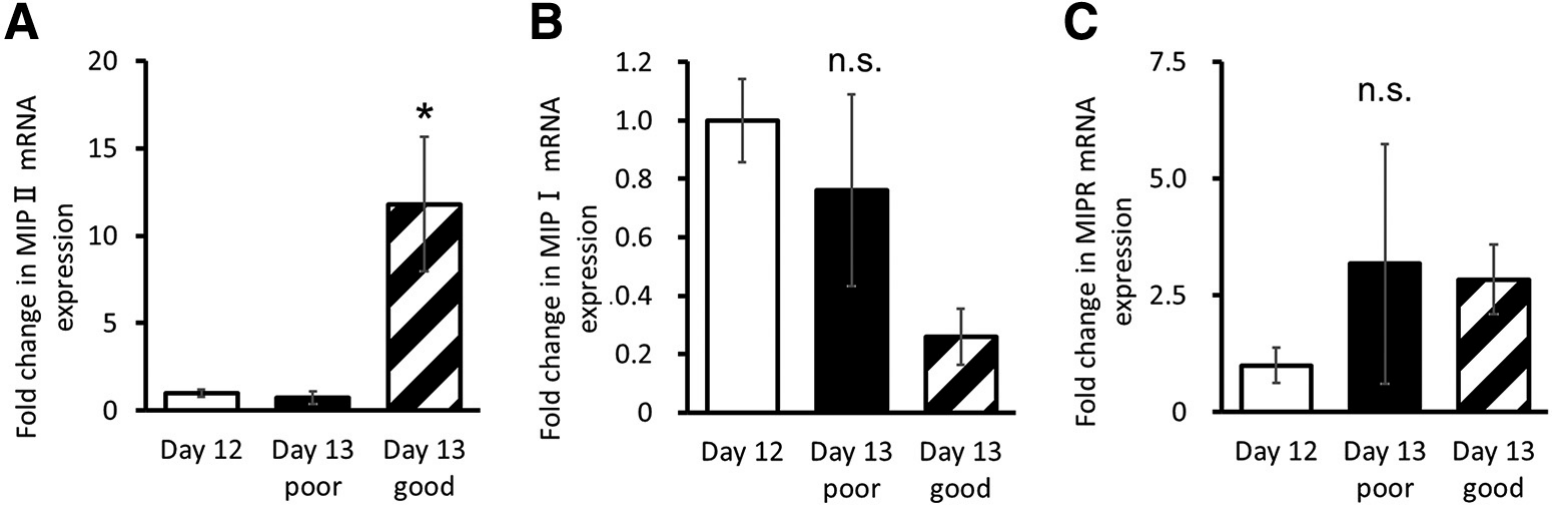
Real-time PCR of MIP II, MIP I, and MIPR mRNA expression in the CNS of day 12 and day 13 good and poor snails. The data were normalized by the mRNA amounts of HSP40 and GPx. ***A***, MIP II mRNA level was significantly increased in day 13 good learner CNS (one-way ANOVA, *n* = 5 each, *F*_(2,12)_ = 7.95, *p *=* *0.0063, Holm *post hoc* test *p =* *0.013 for day 13 good vs day 12; *p *=* *0.0133 for day 13 good vs day 13 poor). ***B***, ***C***, There was no significant difference in the MIP I and MIPR mRNA levels among day 12, day 13 poor learners, and day 13 good learners (one-way ANOVA, *n* = 5 each, MIP I: *F*_(2,12)_ = 3.11, *p *=* *0.081, MIPR: *F*_(2,12)_ = 0.56, *p *=* *0.58). The error bars indicate SEM. n.s., not significant.

To examine the relationship in day 5 snails between the snails that had 1 d of food deprivation that followed 7 d of *ad libitum* feeding (i.e., day 13 snails) and those that did not have the additional day of food deprivation (i.e., day 12 snails), we measured the mRNA expression levels of MIPs and MIPR in the CNS collected from these three groups of snails. Real-time PCR analysis (Fig. 3*A*) showed that there was a significantly higher expression of MIP II mRNA in day 13 good learners (i.e., snails that express LTM) compared with day 12 snails and day 13 poor learners, which did not express LTM (*p *=* *0.013 for day 13 good vs day 12; *p *=* *0.0133 for day 13 good vs day 13 poor). There were no significant differences for MIP I (Fig. 3*B*) or MIPR (Fig. 3*C*; MIP I: *p *=* *0.081, MIPR: *p *=* *0.58) between the three groups of snails (i.e., day 12 vs day 13 snails). The results showing no change in MIPR expression due to CTA training are consistent with the previous data (Hatakeyama et al., 2013). The results above suggest that the ability to retrieve memory in day 13 snails may depend on an increase in insulin levels in the CNS.

### Insulin and the recovery of memory

Insulin modulates synaptic plasticity at synapses between neurons that play key roles in CTA learning and LTM (Murakami et al., 2013b). We hypothesized that in day 5 food-deprived snails the injection of insulin would cause changes in synaptic activity important for CTA learning and memory formation; and as well reduce the hemolymph glucose concentration (Floyd et al., 1999). These changes would mimic the changes seen in day 1 snails, which show the best CTA learning and memory. The changes would also mimic what we believe to be the state of day 13 snails. Day 13 snails learn and form LTM, while day 12 snails do not. Thus, we injected day 12 snails with bovine insulin and then determined whether the insulin-injected day 12 snails retrieve memory. That is, will the insulin injection be equivalent to the 1-d food deprivation experienced in day 13 snails?

**Figure 4.**
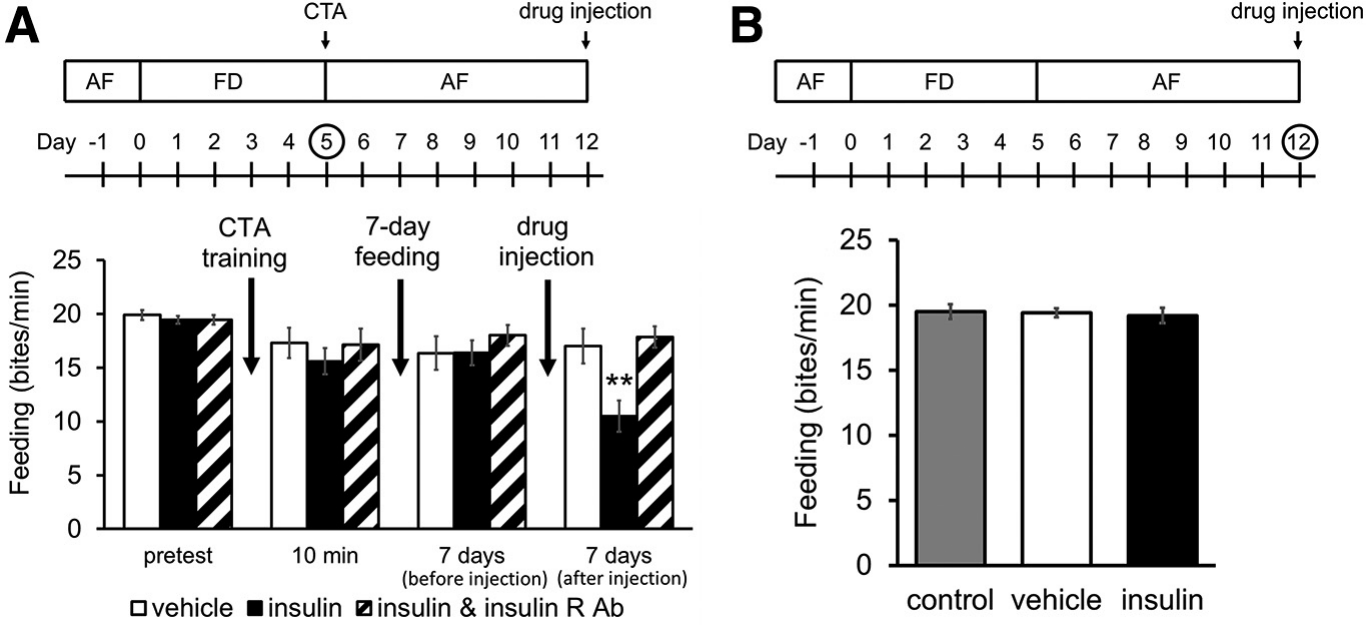
Insulin injection into day 12 snails and LTM. The experimental schedules are shown at the top of the figures. ***A***, Number of bites elicited by the CS in 1 min in the pretest session and the post-test sessions in day 5 snails following 7 d of *ad libitum* access to food and 1 h after drug injections in the day 12 snails. LTM was only expressed in day 12 snails that were injected with bovine insulin. Importantly, note that the simultaneous injection of insulin plus the antibody to the insulin receptor did not allow memory to be recalled (two-way ANOVA, *n* = 40 for insulin injected group and *n* = 20 for vehicle-injected control and simultaneous injection group, *F*_(6,231)_ = 3.51, *p *=* *0.0024 for interaction, *F*_(2,231)_ = 2.97, *p *=* *0.056 for conditioning, *F*_(3,231)_ = 8.70, *p *<* *0.0001 for test, Holm *post hoc* test **p =* *0.0031 for insulin vs vehicle; *p *=* *0.0049 for insulin vs simultaneous injection). ***B***, The insulin injection did not alter feeding behavior in day 12 naive snails (one-way ANOVA, *n* = 10 each, *F*_(2,27)_ = 0.09, *p *=* *0.91). The error bars indicate SEM.

Snails given a 5-d food deprivation followed by CTA training (i.e., day 5 snails) did not show CTA (*p *=* *0.0031 for insulin vs vehicle; *p *=* *0.0049 for insulin vs simultaneous injection; see the data of 10-min post-test in Fig. 4*A*). Then the snails got an *ad libitum* access to food for 7 d (i.e., day 12 snails), and these day 12 snails did not express CTA-LTM (see the data of 7-d post-test in Fig. 4*A*). Following to this protocol, we injected bovine insulin for 1 h to day 12 snails and tested whether the snails expressed CTA-LTM. These snails expressed CTA memory as a phenotype (see the data of 7-d post-test after injection in Fig. 4*A*). We conclude that insulin injection into day 12 snails caused them to perform as day 13 snails. That is, they were able to express CTA-memory.

Further, this insulin-induced expression of memory phenotype was blocked by a simultaneous application of anti-insulin receptor antibody (Fig. 4*A*). We have confirmed that the drug injections did not affect the feeding behaviors in day 12 snails without any conditionings (*p *=* *0.91; Fig. 4*B*). Thus, suppression of feeding responses to the CS seen in day 12 snails receiving the insulin injection was caused by the ability of insulin to alter the internal state of the snails such that the already formed LTM could be recalled (i.e., the CTA-LTM phenotype was observed).

### Change in hemolymph glucose concentration

**Figure 5.**
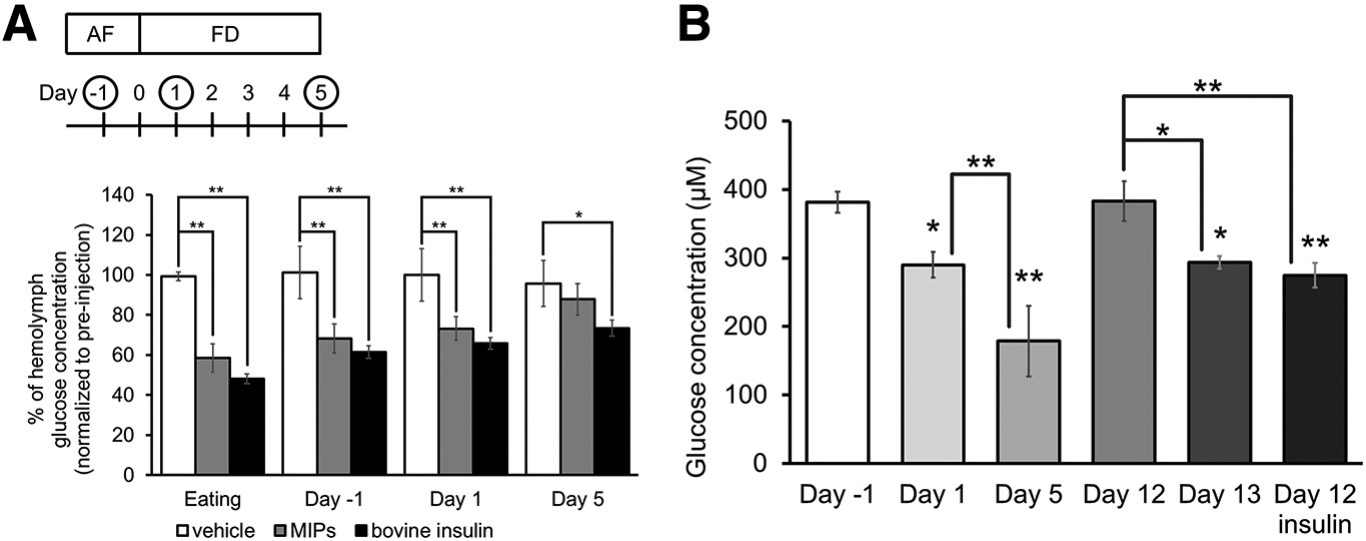
The effects of insulin on hemolymph glucose concentration. ***A***, The experimental procedure is shown above the data. Bovine insulin and MIPs injections significantly reduce hemolymph glucose concentrations in eating, day −1, day 1, and day 5 snails. Notice that MIPs also significantly reduce glucose levels but not on the day 5 snails (two-way factorial ANOVA, *n* = 3 each, *F*_(6,24)_ = 2.38, *p *=* *0.060 for interaction, *F*_(3,24)_ = 6.20, *p *<* *0.0029 for food deprivation, *F*_(2,24)_ = 52.8, *p *<* *0.0001 for treatment, Holm *post hoc* test *p <* *0.05, **p <* *0.01). ***B***, Changes in hemolymph glucose concentration in day −1, day 1, day 5, day 12, day 13, and day 12 snails with an insulin injection. There is a significant difference between glucose levels between day 1 and day 5 snails. There are also significant differences in glucose levels between day 12 and day 13 and day 12 insulin-injected snails (one-way ANOVA, *n* = 10 each, *F*_(5,54)_ = 13.7, *p *<* *0.0001 Holm *post hoc* test *p <* *0.05, **p <* *0.01). The error bars indicate SEM.

Food deprivation in *Lymnaea* alters the hemolymph glucose concentration (Mita et al., 2014a). Here, we compared the glucose concentrations in snails with different durations of food deprivation that were injected with vehicle (HCl, 1.1 μm), partially purified MIPs (50 nm) and bovine insulin (100 nm; Fig. 5). Each of these drugs was injected into the four separate cohorts of snails (eating, day −1, day 1, and day 5). In all the food-deprived states, bovine insulin had a similar effect to MIPs, decreasing the hemolymph glucose concentration in *Lymnaea* (Fig. 5*A*).

We then measured the glucose concentration in hemolymph collected from day 1, day 5, day 12, day 13, and insulin-injected day 12 snails (Fig. 5*B*). Day 5 snails, which indeed learn CTA but which do not express memory after CTA training, had a significantly lower hemolymph glucose concentration (*p *=* *0.0042), compared with day 1 snails, which express CTA memory after training. Day 12 snails, which do not express CTA memory, had a significantly higher hemolymph glucose concentration (*p *=* *0.0185) compared with day 1 snails. Day 13 snails and insulin-injected day 12 snails also had a significantly lower concentration (day 13: *p *=* *0.0193, insulin-injected day 12: *p *=* *0.0053) than day 12 snails. Moreover, the glucose levels in day 13 and day 12 snails injected with insulin were statistically not different and were similar to the level seen in day 1 snails. These data are consistent with the hypothesis that there is an optimal level of circulating glucose that is well correlated with good CTA memory. These data further suggest that 1 d of food deprivation (i.e., day 1 and day 13 snails) results in the optimal levels of insulin and glucose in the snails allowing CTA memory to be recalled.

## Discussion

Together, the data presented here are consistent with the hypothesis that the ability to recall CTA-LTM following CTA training requires both optimal insulin (i.e., MIP II) and glucose levels. These levels can be achieved by a 1-d food-deprivation procedure either occurring initially before CTA training (i.e., day 1 snails) or in day 13 snails. Moreover, we could mimic the results in day 13 snails by the injection of day 12 snails with insulin. Finally, further support of our hypothesis comes from the experiments showing that CTA memory was not recalled when a simultaneous injection of insulin plus its receptor antibody to the insulin receptor occurred.

Previous studies revealed that an insulin spike in the CNS caused by the CS plays an important, and maybe a necessary role, for the CTA-LTM phenotype (Mita et al., 2014a,b). The previous findings are consistent with the data shown here. Only snails with a 1-d food-deprivation procedure (i.e., day 1 and day 13 snails) or day 12 snails injected with insulin express CTA-LTM. In these cohorts, similar insulin and glucose levels are observed. The ability to recall CTA-LTM appears to require a specific internal state of the snail resulting from a release of insulin (insulin spike) caused by a short (1-d) food restriction procedure, coupled to an optimal level of hemolymph glucose.

Insulin signaling is known to modulate synaptic plasticity by acting on glutamatergic and GABAergic receptors (Zhao et al., 2004). Insulin exposure potentiates the activity of NMDA receptor in *Xenopus* oocytes and rat hippocampus (Liu et al., 1995) by recruiting NMDA receptors to the membrane surface (Skeberdis et al., 2001). In cultured differentiating neurons, insulin promotes the functionalization of AMPA synapse (Plitzko et al., 2001). Insulin also causes rapid translocation of the GABA_A_ receptors to the membrane surface in HEK 293 cells transfected with the GABA_A_ receptor (Wan et al., 1997). In addition, insulin also increases plasma membrane expression of glucose transporter-4 (GluT4) in the rat hippocampal neurons during memory formation (Pearson-Leary et al., 2018). It is hypothesized that insulin effects on synaptic plasticity and the increased glucose utilization in neurons by the translocation of receptors and transporters to the plasma membrane are importantly involved in memory formation and its ability to be recalled.

We need to consider the downstream molecular events of what happens when insulin interacts with its receptor (Spinelli et al., 2019). Insulin receptors are tyrosine kinase receptors (Belfiore et al., 2009). Activation of insulin receptor results in activity of the phosphoinositide-3-kinase-protein kinase B/Akt (PI3K/Akt) pathway. This may target molecules such as target of rapamycin (mTOR), glycogen synthase kinase 3 β (GSK3β), and the transcription factors cAMP response element-binding protein (CREB) and the forkhead box O (FoxO) family (Fernandez and Torres-Alemán, 2012). PI3K is activated for glutamate receptor insertion at plasma membranes during synaptic plasticity (Man et al., 2003). mTOR complex 1 (mTORC1) functions for protein synthesis involved in the regulation of long-term synaptic plasticity (Stoica et al., 2011). GSK3β regulates neuroplasticity (Salcedo-Tello et al., 2011). Activation of CREB has been examined in *Lymnaea* and plays a necessary role in LTM formation (Ribeiro et al., 2003; Sadamoto et al., 2004, 2010). Thus, our next study will be focused on the inhibitors for these pathways and molecules.

Presently, we do not understand why the 1-d food-deprivation procedure (i.e., day 1 and day 13 snails) results in an insulin spike. The followings are our speculation. Insulin spike is evoked as an anticipation of food. Snails have been fed daily before CTA training, we may think that this rhythm was learned by snails (Aonuma et al., 2020). Thus, they expected to get fed, and insulin is released in advance, before the food and to an increase in the glucose concentration. In day −1 snails, food arrives and glucose level is kept normal due to the previous release of insulin. However, in day 1 and day 13 snails, there is no food, so the release of insulin may be a “mistake” which leads to a decrease in the glucose level. Not only does this insulin spike alter synaptic transmission in neurons mediating CTA but the spike also has an influence on stabilizing an optimal level of hemolymph glucose. That is, the hemolymph glucose concentrations of day 1 snails, day 13 snails, and day 12 snails with an insulin injection were statistically the same. Only these three cohorts expressed CTA-LTM. Control cohorts injected with vehicle or with insulin and the insulin receptor antibody failed to express LTM. As well, day 12 snails also failed to express CTA-LTM.

Our data show that spontaneous changes in MIP levels occur. Insulin sensitivity in humans is circadian-rhythm dependent (Jarrett and Keen, 1969; Van Cauter et al., 1997; Mattson, 2019). Circadian rhythmicity is also present in *Lymnaea* that affects CTA learning and memory (Wagatsuma et al., 2004), although the rhythmic changes in MIP levels have not yet been examined. Thus, MIP levels can change regardless of food uptake. However, it is important to state that the optimal internal state for CTA is brought by 1-d food deprivation. Further, we should note that a short-term food deprivation makes *Drosophila* the good learners (Hirano and Saitoe, 2013).

We measured the mRNA levels of MIP I (Fig. 3*B*) and MIPR (Fig. 3*C*), and as can be seen in the poor learners, there are large variations in the respective mRNA levels measured compared with those measured in the good learners. There are two main reasons for this. First, these data were obtained from the whole *Lymnaea* CNS. The neurons, such as the light green cells (LGCs) and canopy cells (CCs) in the CNS, are included, and these cells contain MIP mRNAs (Hatakeyama et al., 2000). In addition, the neurons located in the CNS contain MIPR mRNAs ubiquitously (Murakami et al., 2013a). The fact that the mRNA levels vary largely in each individual snail (Fig. 3) means that the function of these neurons varies, resulting in the poor learners. In other words, because the good learners have the stable mRNA levels, the function seems stable. On the other hand, as we hypothesize here, the central state in good learners is dependent on the MIP levels compatible with memory formation. Thus, we would expect that the large variations in mRNA levels exert a bad influence on learning and memory. Second, previous experiments showed that at the electrophysiological level there were large variances in synaptic transmission in poor versus good learners (Kojima et al., 1997). For example, the inhibitory postsynaptic potential recorded in the N1M cells after activation of the cerebral giant cell varied larger in the poor learners than in the good learners after CTA training. Together, these data show that to have memory there must be consistent with the central state (i.e., little variation in MIP levels).

In conclusion, we show here snails express a CTA memory only when an optimal internal state involving an insulin spike and appropriate levels of hemolymph glucose is achieved. It also is apparent that, for example, LTM forms in day 5 snails but cannot be recalled until such time as the necessary insulin spike and optimal glucose hemolymph levels are reached. This may be one of the first indications that an inability to show LTM in an invertebrate model system is not the result of failure to form LTM following training but failure to be able to recall the already formed LTM. That is, if LTM was not already formed, the injection of insulin into day 12 snails or the presence of LTM in day 13 snails (i.e., no further CS-US training occurred but only 1 d of food deprivation) should not have been possible. Thus, we conclude that there is a necessary internal state that is required for the ability to recall CTA-LTM.

## Synthesis

Reviewing Editor: Bong-Kiun Kaang, Seoul National University Decisions are customarily a result of the Reviewing Editor and the peer reviewers coming together and discussing their recommendations until a consensus is reached. When revisions are invited, a fact-based synthesis statement explaining their decision and outlining what is needed to prepare a revision will be listed below. The following reviewer(s) agreed to reveal their identity: Dai Hatakeyama.

Reviewer 1:

I would like to thank the authors for the opportunity to revise the manuscript titled “Induction of LTM following an insulin injection”.

This manuscript tells a good story: it presents solid experimental results, that are well presented and discussed.

I really appreciated the authors’ speculations which clearly discuss their results.

Reviewer 2:

The authors described that even though 5-day food-deprived snails appeared not to learn CTA, they actually learned CTA and expressed their memory if these snails were food-deprived again for one day before the memory test. The authors also showed that such a one-day food deprivation increased the mRNA level of insulin (i.e., MIP). This is so amazing. Before publication, I want to know the following points.

(1) The authors should explain about the internal control genes for qRT-PCR in the section of Materials and Methods, not only the nucleotide sequences of primers shown in the table.

(2) If the snails are food-deprived for 5 days (not 1 day) just before the memory test, what will happen?

(3) In Fig. 3B and C, the error bars of “Day 13 poor” learners were so large, but it converged in “Day 13 good” learners. I want any discussion about this interesting changes.

(4) In Fig. 3C, the authors showed that there was no change in MIPR expression level during CTA. But this data were similar to those by Hatakeyama et al., 2013. The authors should mention this fact.

(5) In Fig. 4A, the authors used anti-insulin receptor antibody for inhibition of snail’s insulin system. Do you have any date mentioning that this antibody recognize and block Lymnaea MIPR?

(6) The present results suggest that insulin level in snails may change spontaneously. Thus, it may change associated with the rhythm inside the body, such as circadian rhythm. The author should discuss the relation between the insulin level and the rhythm inside the body, if possible.

(7) In several graphs (such as Figs. 2A-C, 3B, 3C, 4A and 4B), the titles and the values of y axis are so close.
